# Synthesis of Half-Titanocene Complexes Containing π,π-Stacked Aryloxide Ligands, and Their Use as Catalysts for Ethylene (Co)polymerizations

**DOI:** 10.3390/polym14071427

**Published:** 2022-03-31

**Authors:** Jin Gu, Xiaohua Wang, Wenpeng Zhao, Rui Zhuang, Chunyu Zhang, Xuequan Zhang, Yinghui Cai, Wenbo Yuan, Bo Luan, Bo Dong, Heng Liu

**Affiliations:** 1 Shandong Provincial Key Laboratory of Olefin Catalysis and Polymerization, Chambroad Chemical Industry Research Institute Co., Ltd., Qingdao 266042, China; gujin930118@163.com (J.G.); rui.zhuang@chambroad.com (R.Z.); yinghui.cai@chambroad.com (Y.C.); wenbo.yuan@chambroad.com (W.Y.); bo.luan@chambroad.com (B.L.); 2 Key Laboratory of Rubber-Plastics, Ministry of Education/Shandong Provincial Key Laboratory of Rubber-Plastics, Qingdao University of Science & Technology, Qingdao 266042, China; bh146@qust.edu.cn (X.W.); zwp@qust.edu.cn (W.Z.); cyzhang@qust.edu.cn (C.Z.); xqzhang@qust.edu.cn (X.Z.); 3 Changchun Institute of Applied Chemistry, Chinese Academy of Sciences, Changchun 130022, China

**Keywords:** half-titanocene complexes, π,π-stacking interaction, fused-aryloxide ligands, ethylene (co)polymerization

## Abstract

A family of half-titanocene complexes bearing π,π-stacked aryloxide ligands and their catalytic performances towards ethylene homo-/co- polymerizations were disclosed herein. All the complexes were well characterized, and the intermolecular π,π-stacking interactions could be clearly identified from single crystal X-ray analysis, in which a stronger interaction could be reflected for aryloxides bearing bigger π-systems, e.g., pyrenoxide. Due to the formation of such interactions, these complexes were able to highly catalyze the ethylene homopolymerizations and copolymerization with 1-hexene comonomer, even without any additiveson the aryloxide group, which showed striking contrast to other half-titanocene analogues, implying the positive influence of π,π-stacking interaction in enhancing the catalytic performances of the corresponding catalysts. Moreover, it was found that addition of external pyrene molecules was capable of boosting the catalytic efficiency significantly, due to the formation of a stronger π,π-stacking interaction between the complexes and pyrene molecules.

## 1. Introduction

**Scheme 1:**
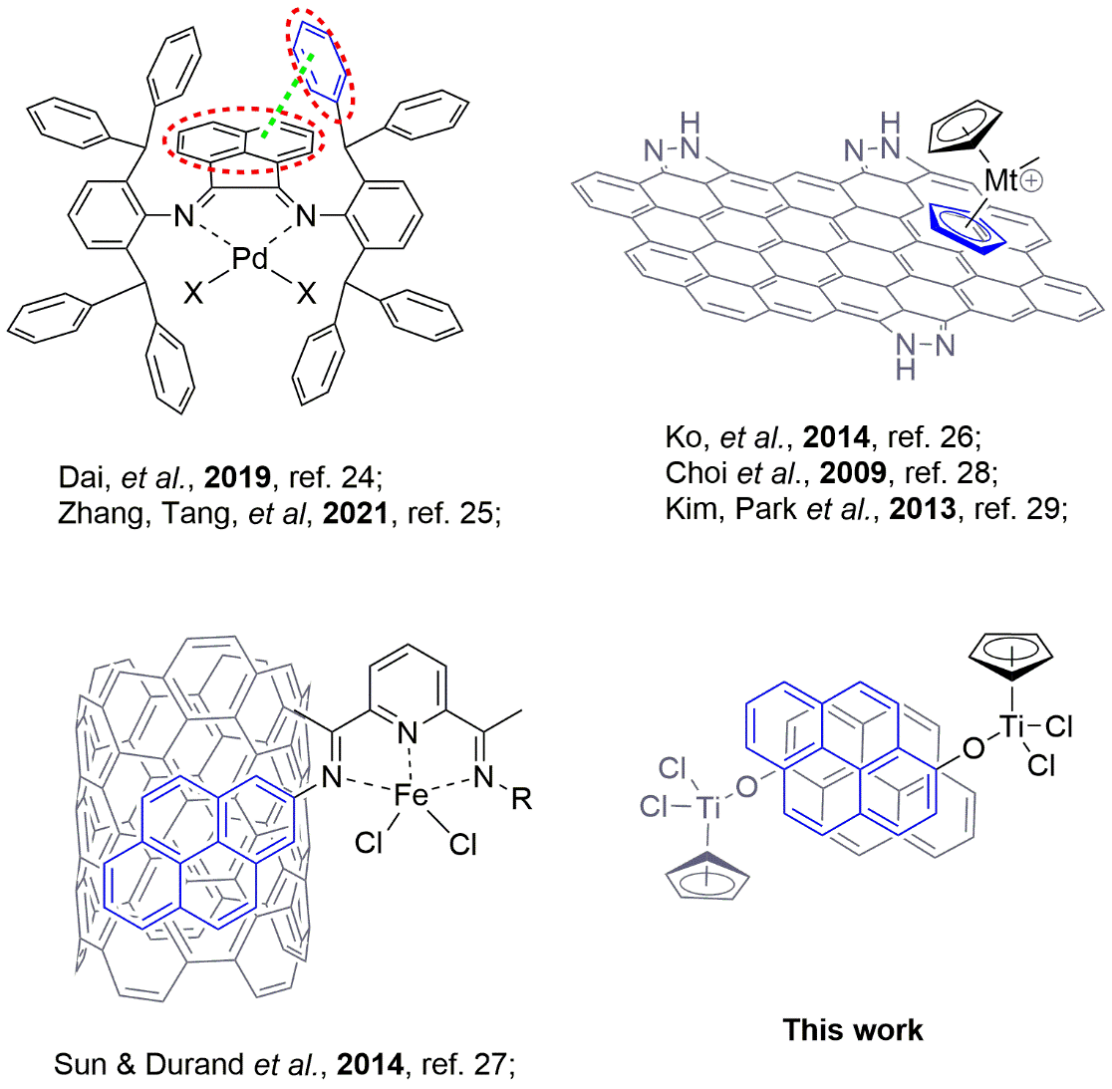
Olefin polymerization catalysts containing π,π-stacking interactions (one of the stacked π-systems is painted into blue to give a clearer distinguishability).

π,π-stacking refers to the *π*-interaction between the π-electron clouds of aromatic systems [1,2]. It is mainly caused by intermolecular overlapping of p orbitals in π-conjugated systems. Based on its stacking patterns, π,π-stacking can be classified into three models: face-to-face (sandwich), edge-to-face (T-shaped), and offset face-to-face (parallel-displaced) [3,4]. Due to its multiplicity and ubiquity, such a non-covalent interaction has been widely explored in many fields of chemistry [5,6,7,8] and biochemistry [9,10,11], and more importantly, it also reveals a decisive role in influencing the course of a reaction [12,13,14,15,16,17,18,19,20,21]. However, regarding olefin polymerizations, the influence of π,π-stacking on catalytic performances is still much less explored. As the field progressed, the main strategy for regulating olefin polymerization behaviors from a catalyst level is still relying on steric and electronic modification of the ligands, and for a long time, scientists have been seeking for effective alternative methodologies [22,23]. Considering its diversity as well as facile construction from simple introducing fused-aryl moieties, π,π-stacking might act as a promising candidate for realizing such a goal, and in recent years, research interest in this field is upsurging (Scheme 1). For instance, incorporation of intra-ligand π,π-interaction is able to improve the thermal robustness of the active species for α-diimine Ni/Pd mediated ethylene (co)polymerizations, and simultaneously regulate the molecular weights and branching densities of the resultant polyethylenes [24,25]; immobilization group IV metallocene and bis(arylimino)pyridine ferrous complexes onto graphene nanoplatelets or carbon nanotubes via *π*,*π*-stacking interactions is capable of enhancing the overall catalytic activities towards olefin polymerization [26,27], and in some cases, affording ultra-high-molecular-weight products, that is difficult to be achieved by traditional catalysts [28,29].

Half-titanocene type complexes Cp’TiX_2_(OR) (Cp’ = substituted cyclopentadienyl; X = Cl, Me etc.; OR = alkyloxide, aryloxide, etc.), is currently one of the most important systems that have been widely explored for ethylene (co)polymerizations [30,31,32,33,34,35,36,37,38,39,40,41,42,43]. In such a system, due to the abundance and commercial availability of diversified phenol derivatives, ethylene (co)polymerization performances as well as the molecular parameters, such as molecular weight, polydispersity, comonomer incorporation percentage, comonomer sequences, etc., can be well regulated through tailoring the substituents on the phenoxide moiety. Based on such considerations, in this research, a series of half-titanocene complexes containing fused-aryloxides ligands were disclosed, and intermolecular π-π stacking interaction can be clearly observed between these aryloxide moieties. Their structural characterizations, as well as the influence of π-π stacking interaction on ethylene homo-/co- polymerization are also studied, which will be given in the following.

## 2. Experimental Section

### 2.1. Materials

All manipulations of air- and moisture-sensitive materials were carried out in a high vacuum line or a glovebox with a medium capacity recirculator (<2 ppm oxygen). The solvents (hexane, toluene, dichloromethane, benzene-*d*^6^) were purchased from Shanghai Aladdin Biochemical Technology Co., Ltd. (Shanghai, China) and refluxed over by sodium or CaH_2_ and degassed by three freeze–pump–thaw cycles prior to use. The Trichloro (cyclopentadienyl) titanium and trichloro (pentamethylcyclopentadienyl) titanium were supplied by Merck Ltd. (Shanghai, China) on Aldrich Chemical Company. DMAO was evaporated under vacuum to obtain a white residue according to the literature [44].

### 2.2. Characterizations

^1^H NMR (400 MHz) and ^13^C NMR (100 MHz) spectra of complexes measured on a Bruker-300 MHz (Bruker Optics, Ettlingen, Germany) in C_6_D_6_ using tetramethylsilane as an internal standard. Ultraviolet−visible (UV−vis) absorption spectra were recorded on a Cary 500 Scan UV−vis spectrophotometer. For the absorption of the UV spectra, the concentration of pyrene was fixed at 5 × 10^−6^ M and the concentration of the host was increased from 0 to 24 × 10^−7^ M in CH_2_Cl_2_ at 298 K. The NMR spectra of the polymers were recorded on a Varian Unity-400 NMR (Varian, Inc., Palo Alto, CA, USA) spectrometer at 135 °C with C_6_D_4_Cl_2_ as a solvent. Elemental analysis was carried out using an elemental Vario EL spectrophotometer (Elementar Analysensysteme GmbH, Langenselbold, Germany). The molecular weights (*M*_n_) and molecular weight distributions (PDI, *M*_w_/*M*_n_) of polymers were determined by PL-GPC 200 high-temperature gel permeation chromatography (Agilent Technologies, CA, USA) at 135 °C using 1,2,4-Trichlorobenzene as an eluent. The melting points of the ethylene/1-hexene copolymers were determined on a TA DSC Q20 instrument (TA, New Castle, DE, USA) at a heating/cooling rate of 10 °C/min. All the DFT calculations were performed with the Gaussian 09 program [45]. The B3LYP functional together with the 6-311+G** basis set for all the atoms. Solvent (toluene) effects were included using the SMD model [46]. The 3D molecular structures displayed in the manuscript were drawn by using CYLview [47].

Crystals of the titanium complexes were obtained by laying hexane onto toluene solutions. Data collections were performed on a Bruker SMART APEX diffractometer at −88.5 °C with a CCD area detector using graphite monochromated MoK radiation (λ = 0.71073 Å). The determination of crystal class and unit cell parameters was carried out by the SMART program package. The raw frame data were processed using SAINT and SADABS to collect the reflection data file. Refinement was performed on F^2^ anistropically for all non-hydrogen atoms by full-matrix least-squares method. Details of X-ray structure determinations and refinements are summarized in Table S1 in the supporting information. CCDC numbers for Ti1 and Ti3: 1874219, 1481991.

### 2.3. Synthesis of Half-Titanocene Complexes

#### 2.3.1. Synthesis of Complex Ti1

A solution of the CpTiCl_3_ (0.5 g, 2.27 mmol) in 10 mL of CH_2_Cl_2_ was reacted with 1.0 *equiv*. of lithium 1-naphthoxide (0.33 g, 2.27 mmol) in 10 mL CH_2_Cl_2_. The mixture was warmed from −78 °C to room temperature and stirred for 12 h. The solvent was evaporated under vacuum to obtain a red residue. The powder was washed twice with diethyl ether (10 mL) and filtered, recrystallization from the concentrated toluene/hexane solution afforded the target complex as red crystals. Yield: 62%. ^1^H NMR (CDCl_3_): δ 8.49–8.47 (m, 1H, Ar-H), 7.57–7.55 (m, 1H, Ar-H), 7.35–7.27 (m, 2H, Ar-H), 7.23–7.19 (m, 1H, Ar-H), 7.09–7.05 (m, 1H, Ar-H), 6.82–6.80 (m, 1H, Ar-H), 6.08 (s, 5H, Cp). ^13^C NMR (126 MHz, CDCl_3_) δ 164.97, 134.48, 127.75, 126.92, 126.70, 125.59, 125.47, 124.66, 122.24, 121.15, 114.66. Anal. Calcd for C_15_H_12_Cl_2_OTi: C, 55.09; H, 3.70. Found: C, 55.29; H, 3.65.

#### 2.3.2. Synthesis of Complex Ti2

The complex Ti2 was carried out using a similar method as preparation of Ti1. Yield: 63%. ^1^H NMR (CDCl_3_): δ 8.59–8.57 (m, 1H, Ar-H), 8.39–8.35 (m, 2H, Ar-H), 7.58–7.33 (m, 5H, Ar-H), 7.20 (s, 1H, Ar-H), 6.07 (s, 5H, Cp). ^13^C NMR (126 MHz, CDCl_3_) δ 163.28, 131.76, 131.18, 128.31, 128.10, 127.68, 127.33, 126.19, 122.97, 122.83, 122.77, 121.24, 119.60, 113.50. Anal. Calcd for C_19_H_14_Cl_2_OTi: C, 60.52; H, 3.74. Found: C, 60.63; H, 3.70.

#### 2.3.3. Synthesis of Complex Ti3

Lithium1-pyrenoxide (0.49 g, 2.27 mmol) was added slowly to a stirred toluene solution (10 mL) containing Cp*TiCl_3_ (0.65 g, 2.27 mmol) at −78 °C. The mixture was warmed to room temperature and then refluxed for 24 h. The red powder was obtained by removing the solvent, recrystallization from the concentrated toluene/hexane solution afforded the desired product as red crystals. Yield: 60%. ^1^H NMR (400 MHz, CDCl_3_, δ, ppm): 8.42–8.40 (m, 1H, Pyrene-H), 8.20–7.85 (m, 7H, Pyrene-H), 7.80–7.71 (m, 1H, Pyrene-H), 6.82 (s, 5H, Cp-H). ^13^C NMR (126 MHz, CDCl_3_) δ 163.45, 131.38, 128.31, 127.13, 126.88, 126.55, 125.64, 125.32, 125.16, 121.17, 120.83, 119.56, 117.21, 103.81. Anal. Calc. for C_21_H_14_Cl_2_OTi (401.1): C, 62.88; H, 3.52. Found: C, 62.91; H, 3.49.

#### 2.3.4. Synthesis of Complex Ti4

The complex Ti4 was carried out using a similar method as preparation of Ti3. Yield: 42%. ^1^H NMR (400 MHz, CDCl_3_, δ, ppm): 8.42–8.40 (m, 1H, Pyrene-H), 8.20–7.85 (m, 7H, Pyrene-H), 7.80–7.71 (m, 1H, Pyrene-H), 1.5 (s, 15H, Cp-Me). ^13^C NMR (126 MHz, CDCl_3_) δ 159.51, 133.24, 131.51, 127.62, 127.28, 126.33, 126.19, 125.57, 125.39, 125.12, 124.84, 121.34, 118.33, 13.13. Anal. Calc. for C_26_H_24_Cl_2_OTi (471.2): C, 66.27; H, 5.13. Found: C, 66.21; H, 4.79.

### 2.4. Polymerization Procedure

A typical polymerization procedure for ethylene polymerization was shown as follows: 100 mL stainless steel autoclave was heated in a vacuum at 80 °C and recharged with ethylene three times, then cooled to room temperature. In a 10 mL Schlenk flask, the additive (pyrene) solution in toluene (1 mL) was added to a solution of titanium complex Ti1, the mixture stirred for 10 min and transferred into the reactor. Then, the required amount of the cocatalyst was added, the autoclave was pressurized to 6 atm immediately. The reaction mixture was stirred at the desired temperature for 10 min. The mixture was then quenched by pouring into a large quantity of acidified ethanol containing HCl (3 M). The polymer was collected by filtration, washed with water and ethanol, and dried to a constant weight under vacuum at 70 °C.

## 3. Results and Discussion

### 3.1. Synthesis and Characterization of the Half-Titanocenes Ti1–Ti4

**Scheme 2:**
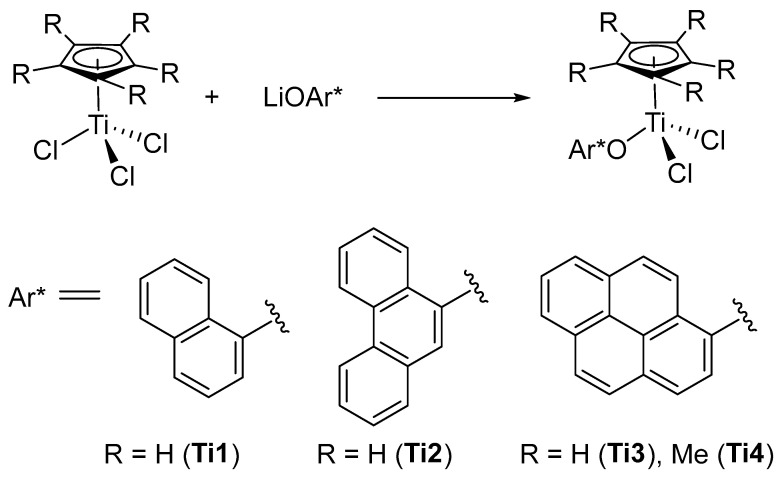
Synthetic procedure for complexes **Ti1**–**Ti4**.

Half-titanocene complexes **Ti1**–**Ti4** containing anionic fused-aryloxide ligands were prepared by stoichiometric reaction between CpTiCl_3_ (or Cp*TiCl_3_) and newly prepared lithium aryloxide derivatives (Scheme 2). Additionally, very pure products could be crystallized as red platelets in high yields upon cooling their saturated n-hexane/toluene solutions to −35 °C in the drybox. In order to establish the structure-activity relationship, fused-aryloxides bearing different π-systems, including 1-naphthoxide, 9-phenanthrenoxide, 1-pyrenoxide, were intentionally explored. All the complexes were well-characterized by NMR and elemental analysis. Moreover, the solid-state structure of **Ti1** and **Ti3** were further confirmed by single crystal X-ray analysis.

**Figure 1:**
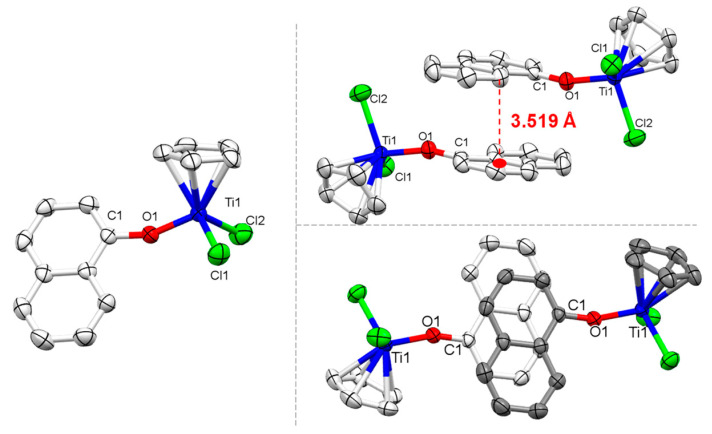
Single crystal structure of complex **Ti1** (left); side-view of the π-π stacked dimer (upper right); top-view of the π-π stacked dimer (lower right, the two naphthalenyl rings were drawn in different color to give a clearer distinguishability). Selected bond length (Å) and angles (°): Ti1-Cl1, 2.2517(10), Ti1-Cl2, 2.2764(10), Ti1-O1, 1.7788(15), O1-C1, 1.365(2), Cl1-Ti1-Cl2, 102.53(5), O1-Ti1-Cl1, 103.76(6), O1-Ti1-Cl2, 102.39(7), C1-O1-Ti1, 158.09(13).

**Figure 2:**
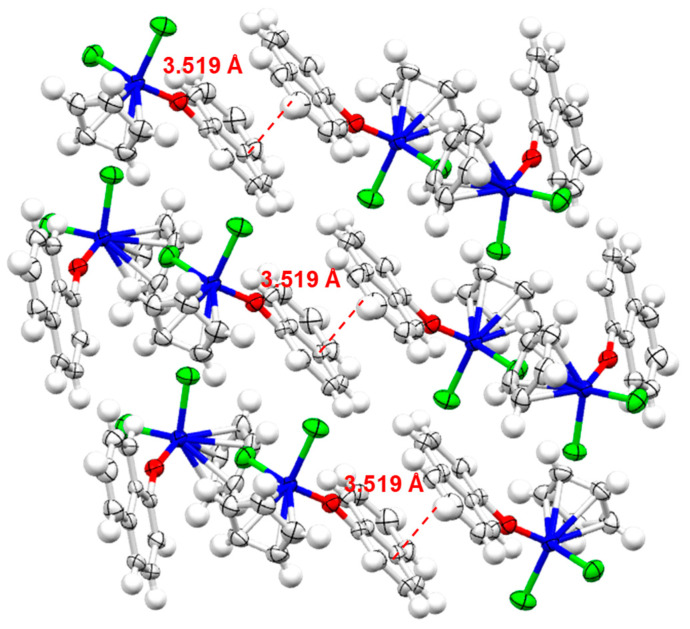
Single crystal packing diagram of complex **Ti1**.

**Figure 3:**
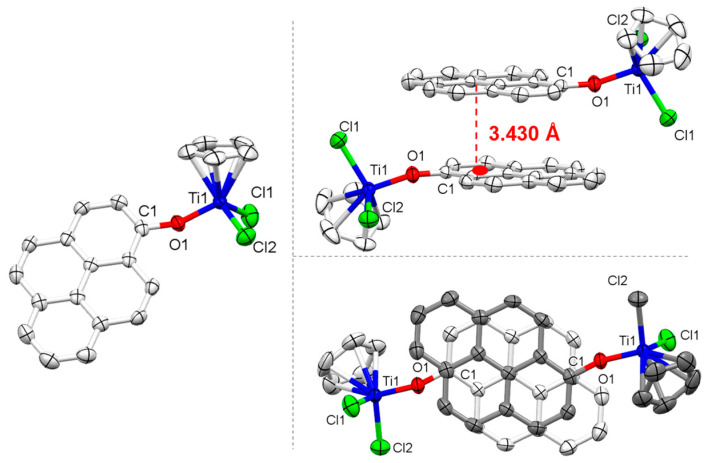
Single crystal structure of complex **Ti3** (**left**); side-view of the π-π stacked dimer (**upper right**); top-view of the π-π stacked dimer (**lower right**, the two pyrenyl rings were drawn in different color to give a clearer distinguishability). Selected bond length (Å) and angles (°): Ti1-O1, 1.7794(18), Ti1-Cl2, 2.2528(8), Ti1-Cl1, 2.2737(9), O1-C1, 1.362(3), O1-Ti1-Cl2, 102.22(7), O1-Ti1-Cl1, 103.00(7), C1-O1-Ti1, 158.26(18).

**Figure 4:**
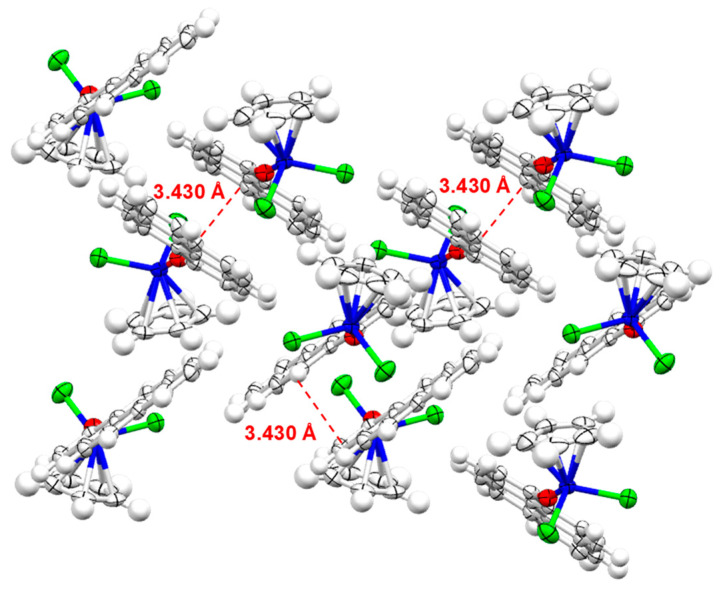
Single crystal packing diagram of complex **Ti3**.

Single crystal structures of complexes **Ti1** and **Ti3** are shown in Figure 1, Figure 2, Figure 3 and Figure 4. In these two complexes, the Ti-O and O-C_ipso_ bond distances are 1.7788(15) Å (**Ti1**), 1.7794(18) Å (**Ti3**) and 1.365(2) Å (**Ti1**), 1.362(3) Å (**Ti3**), respectively, which are quite similar to previously reported CpTiCl_2_(OAr) analogues that reveal Ti-O bond distances of 1.75-1.82 Å and O-C_ipso_ bond distances of ca. 1.36 Å [34,42,48,49,50,51,52,53,54,55,56,57,58]. In contrast, they reveal much larger C_ipso_-O-Ti bond angles (158.09(13)^o^ for **Ti1**, 158.26(18)^o^ for **Ti3**) when comparing half-titanocenes ligated with 2,6-unsubstituted aryloxide moieties that possess similar steric hindrance around the metal center, such as C_ipso_-O-Ti bond angle of 153.77(16)^o^ in CpTiCl_2_(O(4-^t^BuPh) [59]. These larger angles imply much bigger O→Ti π donations into titanium due to the much bigger π systems in fused-aryloxide moieties. Nevertheless, they are still comparatively smaller than counterparts having 2,6-diisopropylphenoxide ligand (163.0(4)^o^ for CpTi, and 173.0(3)^o^ for Cp*Ti) due to the lack of ortho- bulky groups that could ‘sterically’ force the more open C_ipso_-O-Ti angle [31].

As designed, intermolecular π-π stacking interactions can be clearly observed in both two complexes (Figure 1, Figure 2, Figure 3 and Figure 4). Two spatially adjacent anionic fused-aryloxide groups are found to be almost parallel with each other, giving a reversely orientated dimer structure. Additionally, similarly to most cases, an offset stacked conformation was adopted [1,4]. The strength of the π-π stacking interactions can be evaluated by the distances between two almost parallel planes. As illustrated in Figure 2 and Figure 4, an obvious shorter distance with value of 3.430 Å in **Ti3** was observed (versus 3.519 Å in **Ti1**), implying the much stronger π-π interaction in **Ti3**. This result makes sense when considering the overlapping nature of p orbitals in π-conjugated systems, which becomes stronger as the number of π-electrons increases.

### 3.2. Ethylene (Co)polymerization Performances

**Figure 5:**
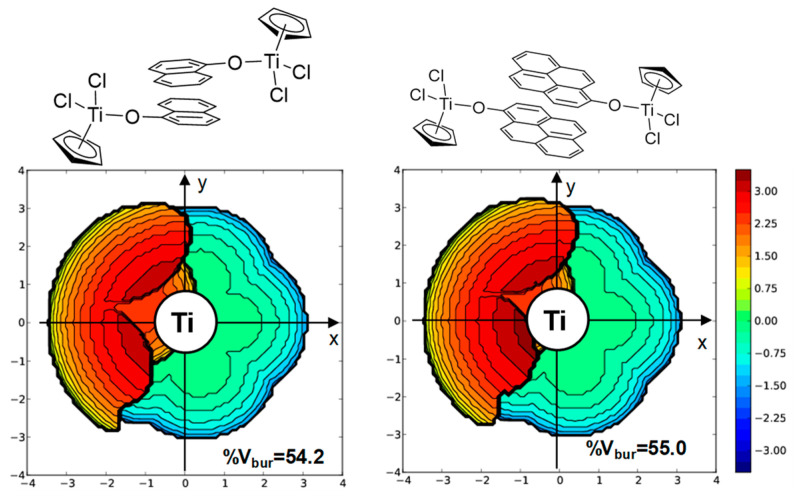
Steric crowding maps from buried volume calculations for π-π stacked **Ti1** (**left**) and **Ti3** (**right**).

Ethylene homopolymerizations were firstly evaluated by using the present half-titanocene complexes **Ti1**–**Ti4** bearing intermolecular π-π stacking interactions. Dry methylaluminoxane (DMAO), which was prepared by removing free trimethylaluminum from commercially available MAO toluene solution [44], was chosen as cocatalyst herein because it had been previously testified to be effective for achieving high catalytic efficiencies as well as high molecular weight products in analogous half-titanocene mediated olefin polymerizations [33]. As the results summarized in Table 1, **Ti1** and **Ti2**, bearing 1-naphthoxide and 9-phenanthrenoxide moieties, respectively, gave very similar catalytic activities of 4.98 × 10^6^ and 5.07 × 10^6^ g PE•mol^−1^ (Ti)•h^−1^; nevertheless, for **Ti3** promoted systems, much lower polymer yields were afforded under identical conditions. Due to the structure similarities of **Ti1** and **Ti3** in Ti-O and O-C_ipso_ bond distances and C_ipso_-O-Ti bond angles that had been concluded from single crystal data, such catalytic differences in **Ti1**, **Ti2** and **Ti3** were probably originated from steric reasons caused by the π-π stacked dimer structure. As the steric crowding maps from buried volume calculations for complexes **Ti1** and **Ti3** (Figure 5) [60,61,62], **Ti3** revealed relative higher buried volume %V_bur_ than **Ti1** (55.0% vs. 54.2%), implying the more sterically crowded environment around the titanium atom, which prevented ethylene monomer from accessing to the metal center and thus eventually resulted in inferior catalytic activities. Additionally, because of the steric congested reason that is able to suppress chain transfer reaction, polyethylene products obtained from **Ti3**/DMAO revealed much higher molecular weight than **Ti1** and **Ti2** mediated systems (M_w_ = 26.2 × 10^4^ g/mol (**Ti1**), 21.8 × 10^4^ g/mol (**Ti2**), 79.4 × 10^4^ g/mol (**Ti3**)).

**Figure 6:**
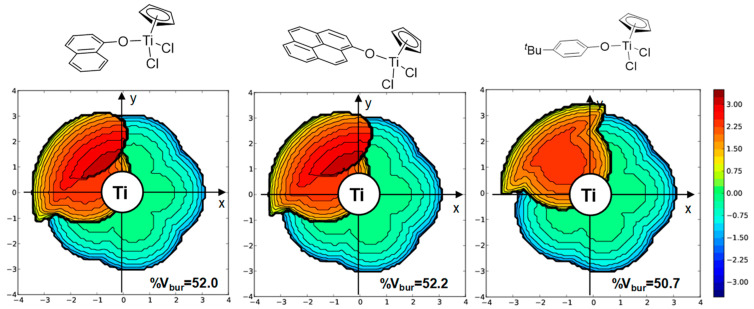
Steric crowding maps from buried volume calculations for unstacked **Ti1** (**left**) and **Ti3** (**middle**) CpTiCl_2_(O(4-^t^BuPh) (**right**).

Another thing worthy of note is that, for half-titanocenes bearing 2,6-unsubstituted aryloxides, such as CpTiCl_2_(O(4-^t^BuPh) and CpTiCl_2_(O(4-MePh), very low catalytic activities generally resulted in olefin polymerization [48]. Such catalytic inefficiencies were probably due to the lack of bulkier ortho-substituents that could force a more open Ti-O-C_ipso_ bond angle, which finally led to less O→Ti donation into Ti atom and therefore destabilized the active species. For the present complexes **Ti1** and **Ti3**; however, although their unstacked structures exhibited similar buried volume %V_bur_ to CpTiCl_2_(O(4-^t^BuPh) (Figure 6, 52.0%, 52.2% vs. 50.7%), appreciable catalytic efficiencies were eventually afforded. These satisfying results were also presumably due to the big π systems caused by π-π stacking interactions, which were able to enhance the electron donation to the metal center and therefore gave a more stable catalytic active species.

The most active precatalyst was concluded to be **Ti4** bearing pentamethylcyclopentadienyl (Cp*) and 1-pyrenoxide ligands, in which a catalytic activity of 5.14 × 10^6^ g PE•mol^−1^ (Ti)•h^−1^ was demonstrated. This was consistent with Nomura’s results that the more electron donating Cp* was able to stabilize the active species, and thus led to higher activity [31].

**Figure 7:**
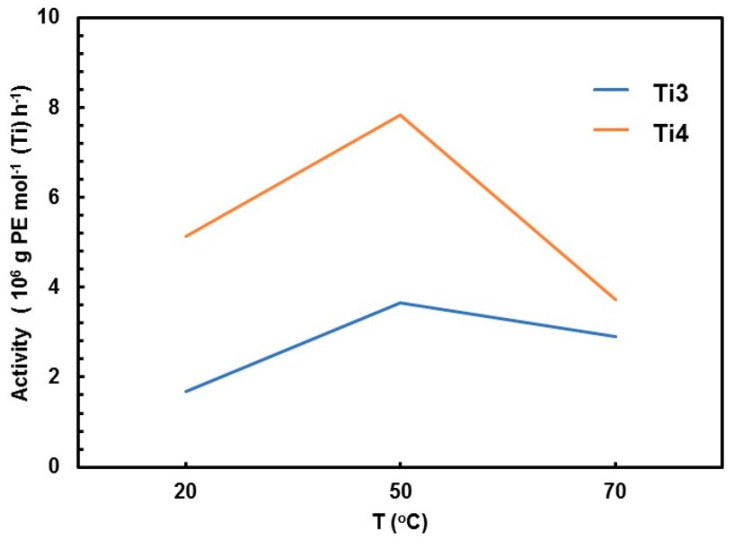
Ethylene polymerization results at various temperatures by **Ti3** and **Ti4**.

π-π stacking conformations are very sensitive to high temperatures. Generally, the stacked dimer structure tends to be dissociated upon increasing the temperature. Therefore, in order to better elucidate the influence of the π system on catalytic performances, ethylene polymerization at different temperatures were carried out by using **Ti3** and **Ti4** at precatalysts. As the data shown in Table 1 and Figure 7, upon increasing the temperature from 20 °C to 70 °C, both of **Ti3** and **Ti4** revealed a first increasing and then decreasing trend, with 50 °C as the optimized temperature. Such increasing polymerization activities from 20 °C to 50 °C were probably due to the dissociation of π-π stacking structures into unstacked active species, which allowed more monomers to access to the metal center, as revealed from the decreased buried volumes %V_bur_ when comparing the stacked and unstacked complexes (55.0% vs. 52.2% for **Ti3**). Further increasing polymerization temperature to 70 °C witnessed obviously decreased activities for both two complexes, which were presumably due to the decomposition of the active species at very high temperatures. Moreover, elevating polymerization temperature also posed big influence on the molecular weights of the resultant polyethylenes. For **Ti3** and **Ti4** mediated polymerizations, a first increasing and then decreasing trend was also observed for the resultant polymer products when increasing the temperature from 20 °C to 70 °C.

**Figure 8:**
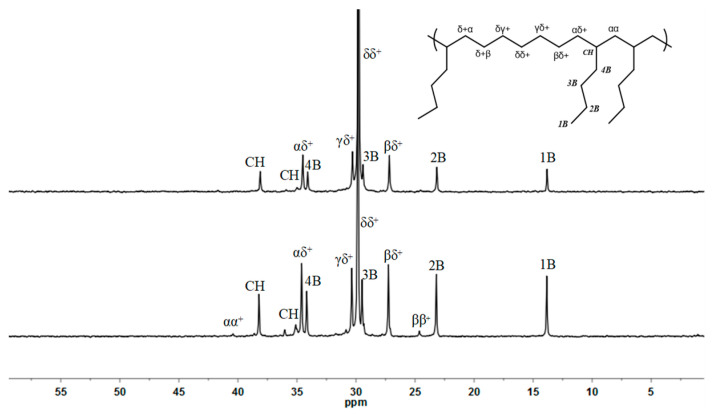
^13^C NMR spectra for ethylene/1-hexene copolymers obtained from entries 11 and 13, Table 2.

Inspired by the good catalytic efficiencies of **Ti3** and **Ti4** towards ethylene polymerization, their performances for copolymerization of ethylene and 1-hexene was also evaluated. As the data summarized in Table 2, moderate to high catalytic activities in the range of 1.08–10.26 × 10^6^ g polymer•mol^−1^ (Ti)•h^−1^ were obtained. Compared to ethylene homopolymerizations, **Ti3** revealed comparable copolymerization activities when 0.32 mol/L comonomer was introduced, further increasing the 1-hexene concentration to 0.48 mol/L resulted in a decreased catalytic activity to 1.08 × 10^6^ g polymer•mol^−1^ (Ti)•h^−1^. In contrast, **Ti4** revealed distinctly different copolymerization behaviors. With an increasing 1-hexene concentration from zero to 0.48 mol/L, **Ti4** demonstrated monotonously increased catalytic activities from 8.19 × 10^6^ g polymer•mol^−1^ (Ti)•h^−1^ to 10.26 × 10^6^ g polymer•mol^−1^ (Ti)•h^−1^. When further increasing 1-hexene concentration to 0.70 mol/L, its activity was hardly changed. The much-improved catalytic activities with increasing 1-hexene concentrations for **Ti4** was probably ascribed to the comonomer effect, which allowed more monomers to access to the active species and thus more enchainment possibilities. Determined by ^13^C NMR (Figure 8), the 1-hexene incorporation levels in the resultant copolymers were in the range of 8.1–15.6%, and comonomer sequence analysis for copolymer samples can be found in Table 3. Determined by DSC analysis, the T_m_ values of the copolymers obtained from **Ti4** decreased gradually from 131 °C to 62 °C, and the DSC curves changed from a sharp peak to broad melting range, indicating the randomly incorporated 1-hexene commoners.

**Scheme 3:**
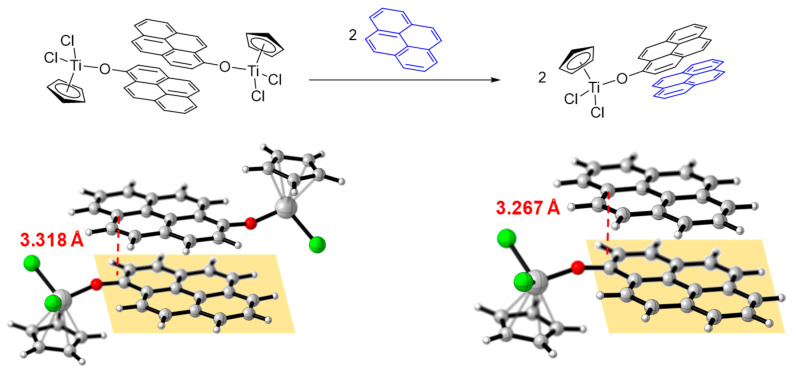
Illustrative scheme for the formation of π,π-stacking between complex **Ti3** and externally added pyrene molecule (structures of π,π-stacked dimer of complex **Ti3** and **Ti3**-pyrene were optimized by DFT calculations and shown in the bottom, one of the pyrenoxide plane was colored into light yellow).

**Figure 9:**
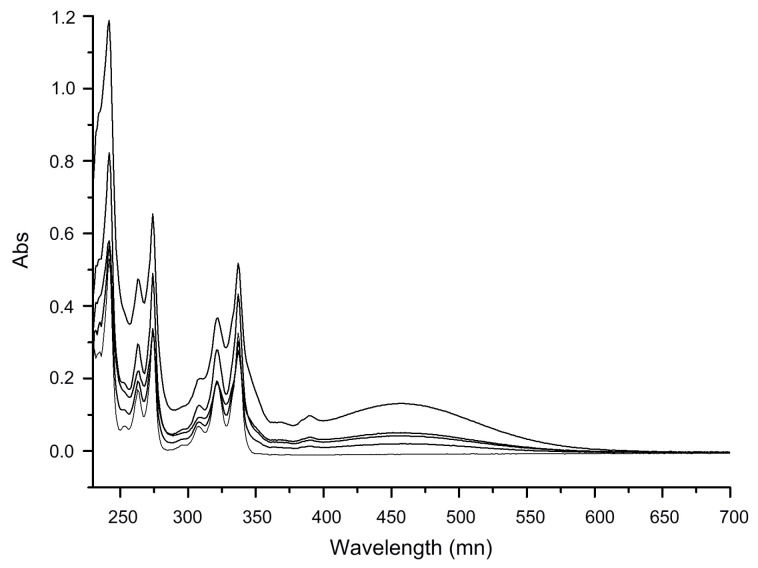
Enhancement in the intensity of the absorption of pyrene with increasing concentration of **Ti3** from 0 to 24 × 10^−7^ M at 25 °C.

**Figure 10:**
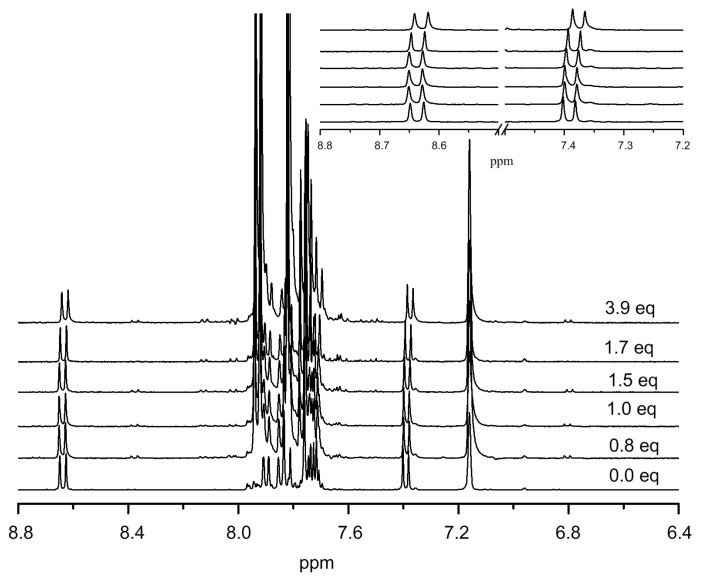
^1^H NMR experiments of gradually adding pyrene to **Ti3** solution in C_6_D_6_.

Considering the positive influence of π,π-stacking on ethylene polymerizations, when comparing with the half-titanocenes counterparts bearing 2,6-unsubstituted aryloxides, we are trying to explore whether externally added π-conjugated small molecules, which will also form π,π-stacking interaction with the fused-aryloxide moieties in the present titanocene complexes, would also enhance the catalytic performances. Based on this, **Ti1**–**Ti3** catalyzed ethylene homopolymerizations were carried out in the presence of 1.0 equiv. of pyrene. As shown by the data in Table 4, obviously enhanced catalytic activities were observed for all the three systems, giving increased values from 4980 to 5190 g PE•mol^−1^ (Ti)•h^−1^ for **Ti1**, from 5070 to 5760 g PE•mol^−1^ (Ti)•h^−1^ for **Ti2**, from 1680 to 2610 g PE•mol^−1^ (Ti)•h^−1^ for **Ti3**, respectively. Moreover, molecular weights of the resultant polyethylenes were also much higher than pyrene-free systems (26.2 × 10^4^ vs. 37.5 × 10^4^ for **Ti1**, 21.8 × 10^4^ vs. 49.3 × 10^4^ for **Ti2**, 79.4 × 10^4^ vs. 111.7 × 10^4^ for **Ti3**), indicating the formed active species therein were more stable and therefore long-lived. These results could be explained by the assumption that the original π,π-stacked dimer of complex **Ti3** would be dissociated in the presence of pyrene molecules and then restack with pyrene to form a more active and stable active species (Scheme 3). Such a speculation could be established after comparing optimized structures of π,π-stacked dimer of complex **Ti3** and **Ti3**-pyrene shown in Scheme 3 (bottom), in which the latter one revealed a relative stronger π,π-stacking interaction than the former one, as evaluated from the distances between two almost parallel planes (3.267 Å vs. 3.318 Å), implying that **Ti3** revealed a bigger tendency to stack with pyrene molecule rather than itself. Such an observation made sense when considering the bigger electron density in pyrene than the pyrenoxide group that was connected to an electrophilic titanium metal center. Because of the same reason, the C_ipso_-O-Ti bond angle of **Ti3**-pyrene was slightly higher than that in **Ti3** dimer (156.9^o^ vs. 156.5^o^). Additionally, the reformation process of π,π-stacking interaction between **Ti3** and pyrene could be also monitored by in situ NMR and UV/Vis studies, which had been reported in other related complexes [15,17,63]. In the UV/Vis experiment, the concentration of **Ti3** was gradually increased from zero to 24 × 10^−7^ M while keeping the concentration of pyrene unchanged (5 × 10^−6^ M). It was found that the intensity of the absorbance of pyrene was gradually enhanced (Figure 9), indicating the formation of strong binding, i.e., π,π-stacking interaction, between **Ti3** and pyrene molecules. This interaction could be also evidenced from the NMR study, which was carried out by gradually adding pyrene (0–3.9 equiv.) to a C_6_D_6_ solution of **Ti3** (17.8 mM). As the spectra shown in Figure 10, two characteristic proton resonance peaks at ca. 8.65 ppm and ca. 7.35 ppm, which were assigned to the 6- and 2- substituted protons on the pyrenoxide group, respectively, witnessed a clear upshift to high field when gradually adding more pyrene molecules. This was due to the formation of a stacking interaction between **Ti3** and pyrene, which caused a bigger shielding effect due to it having a bigger electron density than the pyrenoxide moiety.

## 4. Conclusions

In summary, we have prepared a series of half-titanocene complexes containing fused-aryloxide ligands. Due to the presence of big π-systems therein, such complexes could form π,π-stacking interactions to give dimer structures, and such interactions could be clearly observed from single crystal X-ray spectroscopy analysis. Because of these π,π-stacking interactions, the present half-titanocenes revealed good catalytic activities to ethylene homopolymerizations and copolymerization with 1-hexenes, which confirmed the positive influence of π,π-stacking interaction on enhancing the catalytic performances when comparing with other half-titanocenes bearing 2,6-unsubstituted aryloxide moieties. Moreover, the overall catalytic behaviors of these complexes can be regulated by adding external pyrene additives. Through formation of a stronger π,π-stacking between the complexes and pyrene additives, the catalytic efficiencies as well as the molecular weight of the obtained polymers could be further enhanced.

## Figures and Tables

**Table 1:** Ethylene polymerization with **Ti1**–**Ti4**/DMAO catalytic systems ^a^.

Entry	Cat.	T (°C)	Yield (g)	Activity ^b^	*M*_w_ ^c^ (×10^−4^ g/mol)	*M*_w_/*M*_n_^c^
1	**Ti1**	20	1.66	4980	26.2	1.6
2	**Ti2**	20	1.69	5070	21.8	1.4
3	**Ti3**	20	0.56	1680	79.4	1.8
4	**Ti4**	20	1.82	5146	71.1	2.3
5	**Ti3**	50	1.22	3660	95.1	1.6
6	**Ti3**	70	0.97	2910	60.5	1.4
7	**Ti4**	50	2.61	7830	117.8	1.7
8	**Ti4**	70	1.24	3720	79.7	1.9

^a^ Polymerization: carried out in 60 mL of toluene for 10 min with 2 µmol of titanium catalyst, under an ethylene pressure of 6.0 atm, [Al]/[Ti] = 2000. ^b^ Activity: kg PE•mol-1 (Ti)•h-1. ^c^ Determined by high temperature GPC.

**Table 2:** Ethylene/1-hexene copolymerization with **Ti3** and **Ti4** ^a^.

Entry	Cat	1-hexene (mol/L)	Yield (g)	Activity ^b^	*M*_w_^c^ (×10^−4^ g/mol)	*M*_w_/*M*_n_^c^	Hexene Content (%) ^d^
9	**Ti3**	0.32	0.59	1770	7.1	2.4	8.2
10	**Ti3**	0.48	0.36	1080	7.0	3.0	12.1
11	**Ti4**	0.32	2.73	8190	28.6	2.5	8.1
12	**Ti4**	0.48	3.42	10,260	28.9	2.3	14.0
13	**Ti4**	0.70	3.25	9750	20.8	2.0	15.6

^a^ Polymerization: carried out in 60 mL of toluene for 10 min with 2 µmol of Ti, under an ethylene pressure of 6.0 atm, [DMAO]/[Ti] = 2000, 25 °C. ^b^ Activity: kg polymer•mol-1 (Ti)•h-1. ^c^ Determined by high temperature GPC. ^d^ Determined by NMR.

**Table 3:** Monomer sequence distributions ethylene/1-hexene copolymers obtained with **Ti3** and **Ti4**/DMAO system ^a^.

Cat.	Content (mol%) ^b^	Triad Sequence (%) ^b^						Dyad Sequence (%) ^b^		
		EEE	EEH + HEE	HEH	EHE	EHH + HHE	HHH	EE	EH + HE	HH
**Ti3**	12.1	63.8	16.8	2.3	10.0	2.5	Trace	76.7	21.9	Trace
**Ti4**	14.0	64.4	18.6	2.1	11.0	3.6	Trace	73.7	24.4	Trace

^a^ Polymerization: see Table 2. ^b^ Calculated by ^13^C NMR spectra.

**Table 4:** Ethylene polymerization with **Ti1**-**Ti3**/DMAO system in the presence of 1.0 equiv. of pyrene ^a^.

Entry	Cat.	T (°C)	Yield (g)	Activity ^b^	*M*_w_^c^ (×10^−4^ g/mol)	*M*_w_/*M*_n_^c^
1	**Ti1**	20	1.73	5190	37.5	1.6
2	**Ti2**	20	1.92	5760	49.3	1.6
3	**Ti3**	20	0.87	2610	111.7	1.8

^a^ Polymerization: carried out in 60 mL of toluene for 10 min with 2 µmol of Ti, under an ethylene pressure of 6.0 atm, [Al]/[Ti] = 2000, [pyrene]/[Ti] = 1:1. ^b^ Activity: kg PE•mol-1 (Ti)•h-1. ^c^ Determined by high temperature GPC.

## Supplementary Materials

The following supporting information can be downloaded at: https://www.mdpi.com/article/10.3390/polym14071427/s1, Table S1: Crystallographic data and refinement details for complex Ti1 and Ti3. Figure S1: ^13^C NMR spectrum of ethylene/1-hexene copolymer obtained with Ti1/MAO system (Table 2, Entry 9); Figure S2: ^13^C NMR spectrum of ethylene/1-hexene copolymer obtained with Ti3/MAO system (Table 2, Entry 10); Figure S3: ^13^C NMR spectrum of ethylene/1-hexene copolymer obtained with Ti4/MAO system (Table 2, Entry 11); Figure S4: ^13^C NMR spectrum of ethylene/1-hexene copolymer obtained with Ti4/MAO system (Table 2, Entry 11); Figure S5: ^13^C NMR spectrum of ethylene/1-hexene copolymer obtained with Ti4/MAO system (Table 2, Entry 12).

## References

[1] Zhao Y., Li J., Gu H., Wei D., Xu Y.-C., Fu W., Yu Z. (2015). Conformational Preferences of π–π Stacking Between Ligand and Protein, Analysis Derived from Crystal Structure Data Geometric Preference of π–π Interaction. Interdiscip. Sci..

[2] Hunter C.A., Sanders J.K.M. (1990). The nature of .pi.-.pi. interactions. J. Am. Chem. Soc..

[3] Sutradhar M., Pombeiro A.J.L. (2016). π–π Interaction Directed Applications of Metal Complexes. Non-Covalent Interactions in the Synthesis and Design of New Compounds.

[4] Hunter C.A., Lawson K.R., Perkins J., Urch C.J. (2001). Aromatic interactions. J. Chem. Soc. Perkin Trans. 2.

[5] Despagnet-Ayoub E., Schigand S., Vendier L., Etienne M. (2009). Amine−Phenolate Ligands in Niobium Chemistry: π-Interactions Probed by an Ancillary Alkyne Ligand. Organometallics.

[6] Schultheiss N., Powell D.R., Bosch E. (2003). Silver(I) Coordination Chemistry of 2,6-Diarylpyrazines. π-Stacking, Anion Coordination, and Steric Control. Inorg. Chem..

[7] Tsuzuki S., Honda K., Azumi R. (2002). Model Chemistry Calculations of Thiophene Dimer Interactions:  Origin of π-Stacking. J. Am. Chem. Soc..

[8] Zeni G., Larock R.C. (2004). Synthesis of Heterocycles via Palladium π-Olefin and π-Alkyne Chemistry. Chem. Rev..

[9] Baruah H., Wright M.W., Bierbach U. (2005). Solution Structural Study of a DNA Duplex Containing the Guanine-N7 Adduct Formed by a Cytotoxic Platinum−Acridine Hybrid Agent. Biochemistry.

[10] Zhang X., Lee I., Berdis A.J. (2005). The Use of Nonnatural Nucleotides to Probe the Contributions of Shape Complementarity and π-Electron Surface Area during DNA Polymerization. Biochemistry.

[11] Yan W., Zhang L., Xie D., Zeng J. (2007). Electronic Excitations of Green Fluorescent Proteins:  Modeling Solvatochromatic Shifts of Red Fluorescent Protein Chromophore Model Compound in Aqueous Solutions. J. Phys. Chem. B.

[12] Sabater S., Mata J.A., Peris E. (2014). Catalyst Enhancement and Recyclability by Immobilization of Metal Complexes onto Graphene Surface by Noncovalent Interactions. ACS Catal..

[13] Peris E. (2016). Polyaromatic N-heterocyclic carbene ligands and π-stacking. Catalytic consequences. Chem. Commun..

[14] Ruiz-Botella S., Peris E. (2015). Unveiling the Importance of π-Stacking in Borrowing-Hydrogen Processes Catalysed by Iridium Complexes with Pyrene Tags. Chem. Eur. J..

[15] Ruiz-Botella S., Peris E. (2014). Phenylene- and Biphenylene-Bridged Bis-Imidazolylidenes of Palladium. Influence of the Presence of Pyrene Tags on the Catalytic Activity of the Complexes. Organometallics.

[16] Ibáñez S., Poyatos M., Peris E. (2017). Gold Catalysts with Polyaromatic-NHC ligands. Enhancement of Activity by Addition of Pyrene. Organometallics.

[17] Valdés H., Poyatos M., Ujaque G., Peris E. (2015). Experimental and Theoretical Approaches to the Influence of the Addition of Pyrene to a Series of Pd and Ni NHC-Based Complexes: Catalytic Consequences. Chem. Eur. J..

[18] Peris E. (2018). Smart N-Heterocyclic Carbene Ligands in Catalysis. Chem. Rev..

[19] Liu G., Wu B., Zhang J., Wang X., Shao M., Wang J. (2009). Controlled Reversible Immobilization of Ru Carbene on Single-Walled Carbon Nanotubes: A New Strategy for Green Catalytic Systems Based on a Solvent Effect on π−π Interaction. Inorg. Chem..

[20] Li F., Zhang B., Li X., Jiang Y., Chen L., Li Y., Sun L. (2011). Highly Efficient Oxidation of Water by a Molecular Catalyst Immobilized on Carbon Nanotubes. Angew. Chem. Int. Ed..

[21] Kanao K., Tanabe Y., Miyake Y., Nishibayashi Y. (2010). Intramolecular Edge-to-Face Aromatic π−π Interaction in Optically Active Ruthenium−Allenylidene Complexes for Enantioselective Propargylic Substitution Reactions. Organometallics.

[22] Chen C. (2018). Designing catalysts for olefin polymerization and copolymerization: Beyond electronic and steric tuning. Nat. Rev. Chem..

[23] Li M., Wang R., Eisen M.S., Park S. (2020). Light-mediated olefin coordination polymerization and photoswitches. Org. Chem. Front..

[24] Gong Y., Li S., Tan C., Kong W., Xu G., Zhang S., Liu B., Dai S. (2019). π–π interaction effect in insertion polymerization with α-Diimine palladium systems. J. Catal..

[25] Wang B., Liu H., Zhang C., Tang T., Zhang X. (2021). Propylene homopolymerization and copolymerization with ethylene by acenaphthene-based α-diimine nickel complexes to access EPR-like elastomers. Polym. Chem..

[26] Lee J.S., Ko Y.S. (2014). Synthesis of petaloid graphene/polyethylene composite nanosheet produced by ethylene polymerization with metallocene catalyst adsorbed on multilayer graphene. Catal. Today.

[27] Zhang L., Zhang W., Serp P., Sun W.-H., Durand J. (2014). Ethylene Polymerization Catalyzed by Pyrene-Tagged Iron Complexes: The Positive Effect of π-Conjugation and Immobilization on Multiwalled Carbon Nanotubes. Chemcatchem.

[28] Park S., Choi I.S. (2009). Production of Ultrahigh-Molecular-Weight Polyethylene/Pristine MWCNT Composites by Half-Titanocene Catalysts. Adv. Mater..

[29] Choi B., Lee J., Lee S., Ko J.-H., Lee K.-S., Oh J., Han J., Kim Y.-H., Choi I.S., Park S. (2013). Generation of Ultra-High-Molecular-Weight Polyethylene from Metallocenes Immobilized onto N-Doped Graphene Nanoplatelets. Macromol. Rapid. Comm..

[30] Nomura K. (2009). Half-titanocenes containing anionic ancillary donor ligands as promising new catalysts for precise olefin polymerisation. Dalton Trans..

[31] Nomura K., Liu J.Y., Padmanabhan S., Kitiyanan B. (2007). Nonbridged half-metallocenes containing anionic ancillary donor ligands: New promising candidates as catalysts for precise olefin polymerization. J. Mol. Catal. A Chem..

[32] Redshaw C., Tang Y. (2012). Tridentate ligands and beyond in group IV metal α-olefin homo-/co-polymerization catalysis. Chem. Soc. Rev..

[33] Nomura K., Fukuda H., Apisuk W., Trambitas A.G., Kitiyanan B., Tamm M. (2012). Ethylene copolymerization by half-titanocenes containing imidazolin-2-iminato ligands–MAO catalyst systems. J. Mol. Catal. A Chem..

[34] Nomura K., Tanaka A., Katao S. (2006). Effect of aryloxide ligand in 1-hexene, styrene polymerization catalyzed by nonbridged half-titanocenes of the type, Cp’TiCl2(OAr) (Cp′=C5Me5, tBuC5H4): Structural analyses for Cp*TiCl2(O-2,6-tBu2C6H3) and Cp*TiCl2(O-2,6-iPr2-4-tBuC6H2). J. Mol. Catal. A Chem..

[35] Nomura K., Fukuda H., Matsuda H., Katao S., Patamma S. (2015). Synthesis and structural analysis of half-titanocenes containing 1,3-imidazolidin-2-iminato ligands: Effect of ligand substituents in ethylene (co)polymerization. J. Organomet. Chem..

[36] Nomura K., Yamada J., Wang W., Liu J. (2007). Effect of ketimide ligand for ethylene polymerization and ethylene/norbornene copolymerization catalyzed by (cyclopentadienyl) (ketimide)titanium complexes–MAO catalyst systems: Structural analysis for Cp∗TiCl2(NCPh2). J. Organomet. Chem..

[37] Nomura K., Liu J., Fujiki M., Takemoto A. (2007). Facile, Efficient Functionalization of Polyolefins via Controlled Incorporation of Terminal Olefins by Repeated 1,7-Octadiene Insertion. J. Am. Chem. Soc..

[38] Harakawa H., Patamma S., Boccia A.C., Boggioni L., Ferro D.R., Losio S., Nomura K., Tritto I. (2018). Ethylene Copolymerization with 4-Methylcyclohexene or 1-Methylcyclopentene by Half-Titanocene Catalysts: Effect of Ligands and Microstructural Analysis of the Copolymers. Macromolecules.

[39] Kim T.-J., Kim S.-K., Kim B.-J., Hahn J.S., Ok M.-A., Song J.H., Shin D.-H., Ko J., Cheong M., Kim J. (2009). Half-Metallocene Titanium(IV) Phenyl Phenoxide for High Temperature Olefin Polymerization: Ortho-Substituent Effect at Ancillary o-Phenoxy Ligand for Enhanced Catalytic Performance. Macromolecules.

[40] Kim T.-J., Kim S.-K., Kim B.-J., Son H.-J., Hahn J.S., Cheong M., Mitoraj M., Srebro M., Piękoś Ł., Michalak A. (2010). Sterically Less-Hindered Half-Titanocene(IV) Phenoxides: Ancillary-Ligand Effect on Mono-, Bis-, and Tris(2-Alkyl-/arylphenoxy) Titanium(IV) Chloride Complexes. Chem. Eur. J..

[41] Kitphaitun S., Yan Q., Nomura K. (2020). The Effect of SiMe3 and SiEt3 Para Substituents for High Activity and Introduction of a Hydroxy Group in Ethylene Copolymerization Catalyzed by Phenoxide-Modified Half-Titanocenes. Angew. Chem. Int. Ed..

[42] Dong B., Zhang H., Li H., Liu H., Guo J., Zhang C., Zhang X., Hu Y., Sun G., Zhang X. (2016). Half-titanocene complexes bearing bulky dibenzhydryl-substituted aryloxide ligand: Syntheses, characterization, and ethylene (Co-)polymerization behaviors. Polymer.

[43] Thakur A., Baba R., Chammingkwan P., Terano M., Taniike T. (2018). Synthesis of aryloxide-containing half-titanocene catalysts grafted to soluble polynorbornene chains and their application in ethylene polymerization: Integration of multiple active centres in a random coil. J. Catal..

[44] Nishii K., Matsumae T., Dare E.O., Shiono T., Ikeda T. (2004). Effect of Solvents on Living Polymerization of Propylene with [t-BuNSiMe2Flu]TiMe2-MMAO Catalyst System. Macromol. Chem. Phys..

[45] Frisch M., Trucks G., Schlegel H., Scuseria G., Robb M., Heeseman J., Scalmani G., Barone V., Mennucci B., Petersson G. (2009). Gaussian 09, rev. A.02.

[46] Marenich A.V., Cramer C.J., Truhlar D.G. (2009). Universal Solvation Model Based on Solute Electron Density and on a Continuum Model of the Solvent Defined by the Bulk Dielectric Constant and Atomic Surface Tensions. J. Phys. Chem. B.

[47] Legault C.Y. (2009). CYLview, ver. 1.0.

[48] Nomura K., Naga N., Miki M., Yanagi K. (1998). Olefin Polymerization by (Cyclopentadienyl)(aryloxy)titanium(IV) Complexes−Cocatalyst Systems. Macromolecules.

[49] Nomura K., Naga N., Miki M., Yanagi K., Imai A. (1998). Synthesis of Various Nonbridged Titanium(IV) Cyclopentadienyl−Aryloxy Complexes of the Type CpTi(OAr)X2 and Their Use in the Catalysis of Alkene Polymerization. Important Roles of Substituents on both Aryloxy and Cyclopentadienyl Groups. Organometallics.

[50] Thorn M.G., Vilardo J.S., Lee J., Hanna B., Fanwick P.E., Rothwell I.P. (2000). Synthesis, Characterization, and One-Electron Reduction of Mixed-Cyclopentadienyl/Aryloxide Titanium Dichlorides. Organometallics.

[51] Thorn M.G., Vilardo J.S., Fanwick P.E., Rothwell I.P. (1998). Formation and reactivity of cationic alkyl derivatives of titanium containing ortho-(1-naphthyl)phenoxide ligation. Chem. Commun..

[52] Arévalo S., Bonillo M.R., de Jesús E., de la Mata F.J., Flores J.C., Gómez R., Gómez-Sal P., Ortega P. (2003). Synthesis of polymetallic Group 4 complexes bridged by benzenediolate and triolate ligands. X-ray crystal structure of [{Ti(C5Me5)Cl2}2{μ-1,4-O(2,3-C6H2Me2)O}]. J. Organomet. Chem..

[53] Arévalo S., de Jesús E., de la Mata F.J., Flores J.C., Gómez R., Gómez-Sal M.P., Ortega P., Vigo S. (2003). Synthesis of Aryloxo Cyclopentadienyl Group 4 Metal-Containing Dendrimers. Organometallics.

[54] Turner L.E., Thorn M.G., Swartz Ii R.D., Chesnut R.W., Fanwick P.E., Rothwell I.P. (2003). Synthesis, stereochemistry, bonding and fluxionality of 2-(inden-3-yl)phenols and their cyclopentadienyl titanium derivatives. Dalton Trans..

[55] Sturla S.J., Buchwald S.L. (2002). Monocyclopentadienyltitanium Aryloxide Complexes:  Preparation, Characterization, and Application in Cyclization Reactions. Organometallics.

[56] Firth A.V., Stephan D.W. (1998). Monocyclopentadienyl−Titanium Aryloxide Sulfide Complexes. Inorg. Chem..

[57] Gómez-Sal P., Martín A., Mena M., Royo P., Serrano R. (1991). Monopentamethylcyclopentadienyltitanium(IV) halo-alkoxides, alkyl-alkoxides and acetylacetonates. J. Organomet. Chem..

[58] Schröder K., Haase D., Saak W., Beckhaus R., Kretschmer W.P., Lützen A. (2008). Tetrabenzo[a,c,g,i]fluorenyltitanium(III) and -(IV) Complexes: Syntheses, Reactions, and Catalytic Application. Organometallics.

[59] Nielson A.J., Harrison J.A., Shen C., Waters J.M. (2006). Steric influences in cyclopentadienyl-monophenoxide complexes of titanium(iv) arising from ortho-substitution of the phenoxide ligand. Polyhedron.

[60] Falivene L., Cavallo L., Talarico G. (2015). Buried Volume Analysis for Propene Polymerization Catalysis Promoted by Group 4 Metals: A Tool for Molecular Mass Prediction. ACS Catal..

[61] Falivene L., Credendino R., Poater A., Petta A., Serra L., Oliva R., Scarano V., Cavallo L. (2016). SambVca 2. A Web Tool for Analyzing Catalytic Pockets with Topographic Steric Maps. Organometallics.

[62] Falivene L., Cao Z., Petta A., Serra L., Poater A., Oliva R., Scarano V., Cavallo L. (2019). Towards the online computer-aided design of catalytic pockets. Nat. Chem..

[63] Zhang W.-Y., Han Y.-F., Weng L.-H., Jin G.-X. (2014). Synthesis, Characterization, and Properties of Half-Sandwich Iridium/Rhodium-Based Metallarectangles. Organometallics.

