# Supplementary material for: Synthesis of Half-Titanocene Complexes Containing π,π-Stacked Aryloxide Ligands, and Their Use as Catalysts for Ethylene (Co)polymerizations
Source: Polymers (Basel). 2022 Mar 31;14(7):1427. doi: 10.3390/polym14071427 (PMC9002825; doi:10.3390/polym14071427)
Supplement: Supplementary file 1 [file polymers-14-01427-s001.zip › supporting information.pdf]

Synthesis of Half-Titanocene Complexes Containing π,π-Stacked Aryloxide Ligands, and Their Use as Catalysts for Ethylene (Co)polymerizations

Jin Gu,[1] Xiaohua Wang,[2] Wenpeng Zhao,[2] Rui Zhuang,[1,3] Chunyu Zhang,[2,3]Xuequan Zhang[2,3], Yinghui Cai[1], Wenbo Yuan[1], Bo Luan[1], Bo Dong,[1,3*] Heng Liu[2,3*]

[1] Shandong Provincial Key Laboratory of Olefin Catalysis and Polymerization, Chambroad Chemical Industry Research Institute Co., Ltd.，Qingdao, 266042, China;
[2] Key Laboratory of Rubber-Plastics, Ministry of Education/ Shandong Provincial Key Laboratory of Rubber-plastics, Qingdao University of Science & Technology, Qingdao, 266042, China;
[3] Changchun Institute of Applied Chemistry, Chinese Academy of Sciences, Changchun, 130022, Jilin, China;

Contents

1. Crystal data and structure refinement detials table for **Ti1**,**Ti3** S**2**

2. NMR Figures of poly(ethylene/1-hexene) obtained in different conditions S**3**

Table S1 Crystallographic data and refinement details for complex **Ti1** and **Ti3**.

	**Ti1**	**Ti3**
Empirical formula	$C_{15}H_{12}Cl_2OTi$	$C_{22}H_{16}Cl_4OTi$
Fomula weight	327.05	486.05
Crystal color	Red	Red
Temperature (K)	273	187
Wave length (Å)	0.71073	0.71073
Crystal system	Monoclinic	Monoclinic
Space group	$P2_1/c$	$P2_1/c$
a (Å)	9.989 (6)	16.662(12)
b (Å)	7.039 (4)	10.291 (10)
c (Å)	19.978 (11)	12.087 (4)
α (°)	90	90
β (°)	92.25	101.1 (2)
γ (°)	90	90
Volume($Å^3$)	1403.6	2033.6
Z	4	4
D calcd (mg m^{-3})	1.548	1.588
μ (mm^{-1})	0.98	0.96
F (000)	664	984
Crystal size (mm)	0.17 × 0.16 × 0.15	0.21 × 0.18 × 0.11
θ range (°)	2.8–26.4	2.3–26.4
Limiting indices	$-12 \leq h \leq 12$	$-20 \leq h \leq 20$
	$-8 \leq k \leq 8$	$-12 \leq k \leq 12$
	$-13 \leq l \leq 24$	$-8 \leq l \leq 15$
No. of rflns collected	2848	12457
No. of unqiue rflns	2567	4135
R_{int}	0.037	0.029
Goodness-of-fit on F^2	1.05	1.03
*w*R2	0.091	0.122

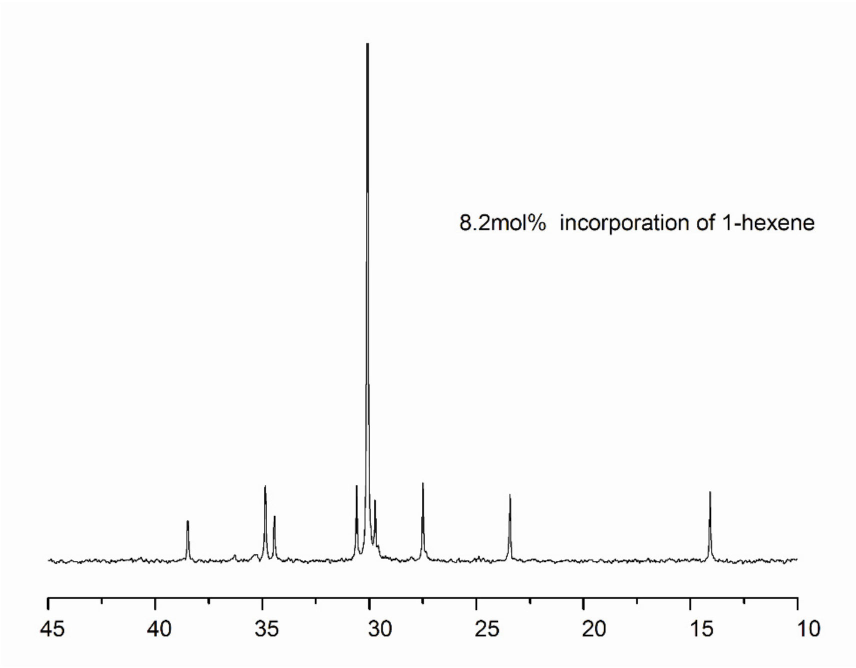

Figure S1 ^{13}C NMR spectrum of ethylene/1-hexene copolymer obtained with **1a**/MAO system (**Table 2, Entry 9**).

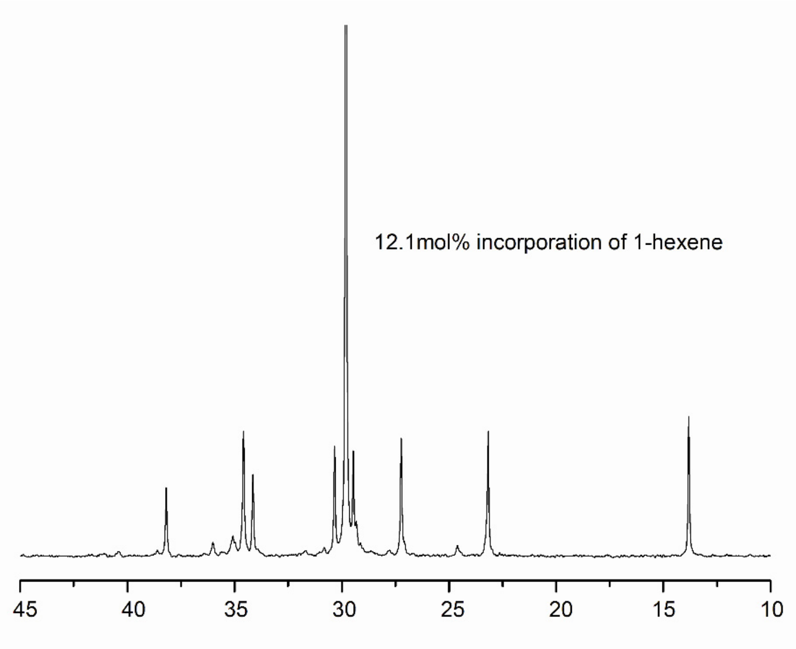

Figure S2 ^{13}C NMR spectrum of ethylene/1-hexene copolymer obtained with **1a**/MAO system (**Table 2, Entry 10**).

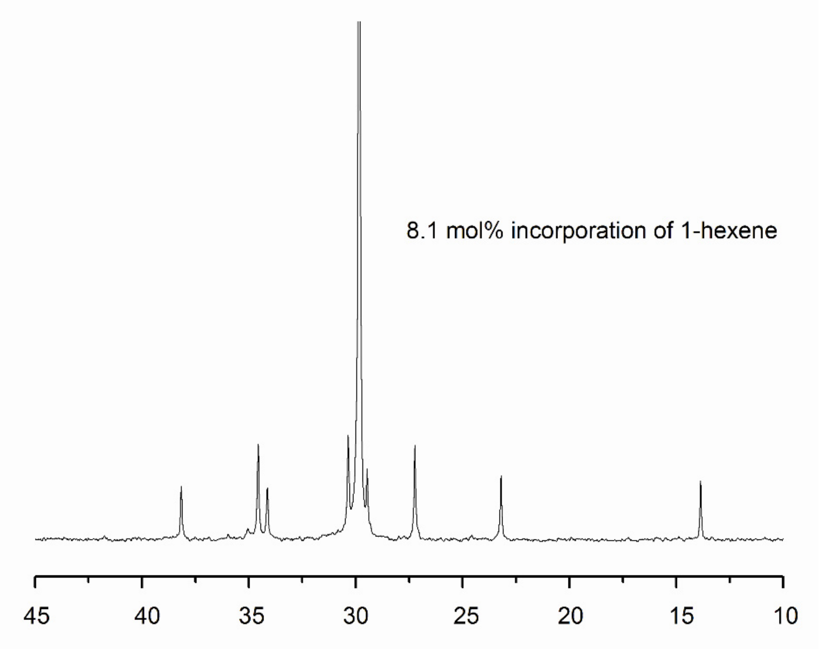

Figure S3 ^{13}C NMR spectrum of ethylene/1-hexene copolymer obtained with **2a**/MAO system (**Table 2, Entry 11**).

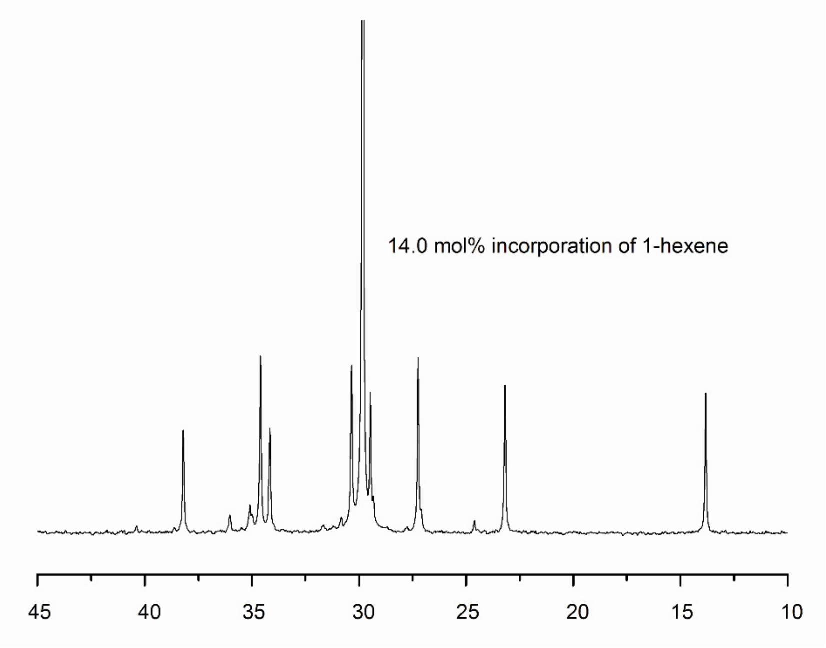

Figure S4. ^{13}C NMR spectrum of ethylene/1-hexene copolymer obtained with **2a**/MAO system (**Table 2, Entry 11**).

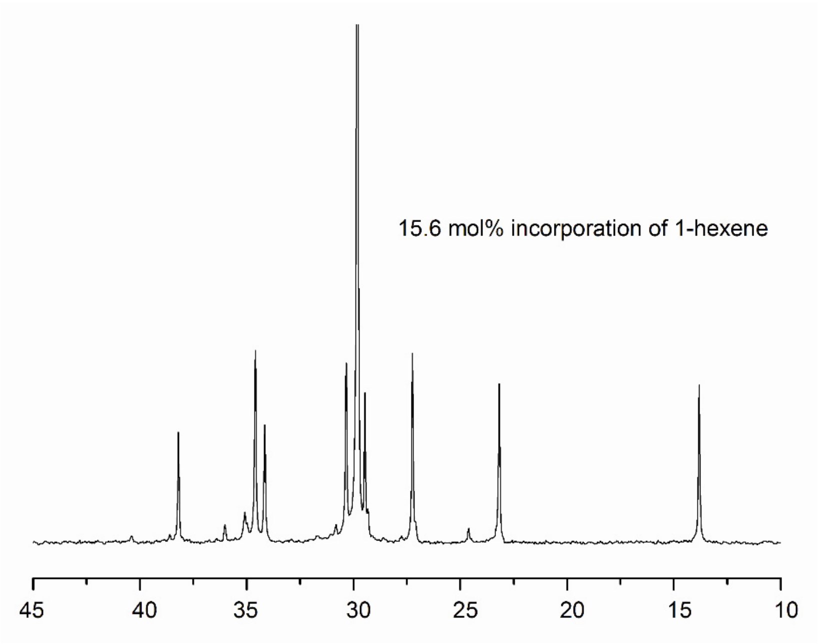

Figure S5 ^{13}C NMR spectrum of ethylene/1-hexene copolymer obtained with **2a**/MAO system (**Table 2, Entry 12**).
