# Supplementary material for: Synthesis of Half-Titanocene Complexes Containing π,π-Stacked Aryloxide Ligands, and Their Use as Catalysts for Ethylene (Co)polymerizations
Source: Polymers (Basel). 2022 Mar 31;14(7):1427. doi: 10.3390/polym14071427 (PMC9002825; doi:10.3390/polym14071427)

checkCIF/PLATON report

You have not supplied any structure factors. As a result the full set of tests cannot be run.

THIS REPORT IS FOR GUIDANCE ONLY. IF USED AS PART OF A REVIEW PROCEDURE FOR PUBLICATION, IT SHOULD NOT REPLACE THE EXPERTISE OF AN EXPERIENCED CRYSTALLOGRAPHIC REFEREE.

No syntax errors found.   CIF dictionary   Interpreting this report

Datablock: a064

```
Bond precision:  C-C = 0.0040 A          Wavelength=0.71073

Cell:           a=16.6628(12)    b=10.2910(8)       c=12.0875(9)
                alpha=90         beta=101.154(1)    gamma=90
Temperature:    187 K

                Calculated                Reported
Volume          2033.6(3)                 2033.6(3)
Space group     P 21/c                    P2(1)/c
Hall group      -P 2ybc                   ?
Moiety formula  C21 H14 Cl2 O Ti, C H2 Cl2 ?
Sum formula     C22 H16 Cl4 O Ti          C22 H16 Cl4 O Ti
Mr              486.02                    486.05
Dx,g cm-3       1.587                     1.588
Z               4                         4
Mu (mm-1)       0.957                     0.957
F000            984.0                     984.0
F000'           987.75
h,k,lmax        20,12,15                  20,12,15
Nref            4148                      4135
Tmin,Tmax       0.818,0.900               0.824,0.902
Tmin'           0.818

Correction method= # Reported T Limits: Tmin=0.824 Tmax=0.902
AbsCorr = MULTI-SCAN

Data completeness= 0.997          Theta(max)= 26.360

R(reflections)= 0.0454( 3462)     wR2(reflections)= 0.1238( 4135)

S = 1.033                  Npar= 253
```

The following ALERTS were generated. Each ALERT has the format
test-name_ALERT_alert-type_alert-level.
Click on the hyperlinks for more details of the test.

Alert level C

```
PLAT242_ALERT_2_C Low    'MainMol' Ueq as Compared to Neighbors of        Ti1 Check
```

Alert level G

```
PLAT005_ALERT_5_G No Embedded Refinement Details found  in the CIF     Please Do !
PLAT066_ALERT_1_G Predicted and Reported Tmin&Tmax Range Identical          ? Check
PLAT899_ALERT_4_G SHELXL97   is Deprecated and Succeeded by SHELXL       2014 Note
```

```
   0 ALERT level A = Most likely a serious problem - resolve or explain
   0 ALERT level B = A potentially serious problem, consider carefully
   1 ALERT level C = Check. Ensure it is not caused by an omission or oversight
   3 ALERT level G = General information/check it is not something unexpected

   1 ALERT type 1 CIF construction/syntax error, inconsistent or missing data
   1 ALERT type 2 Indicator that the structure model may be wrong or deficient
   0 ALERT type 3 Indicator that the structure quality may be low
   1 ALERT type 4 Improvement, methodology, query or suggestion
   1 ALERT type 5 Informative message, check
```

It is advisable to attempt to resolve as many as possible of the alerts in all categories. Often the minor alerts point to easily fixed oversights, errors and omissions in your CIF or refinement strategy, so attention to these fine details can be worthwhile. In order to resolve some of the more serious problems it may be necessary to carry out additional measurements or structure refinements. However, the purpose of your study may justify the reported deviations and the more serious of these should normally be commented upon in the discussion or experimental section of a paper or in the "special_details" fields of the CIF. checkCIF was carefully designed to identify outliers and unusual parameters, but every test has its limitations and alerts that are not important in a particular case may appear. Conversely, the absence of alerts does not guarantee there are no aspects of the results needing attention. It is up to the individual to critically assess their own results and, if necessary, seek expert advice.

Publication of your CIF in IUCr journals

A basic structural check has been run on your CIF. These basic checks will be run on all CIFs submitted for publication in IUCr journals (*Acta Crystallographica*, *Journal of Applied Crystallography*, *Journal of Synchrotron Radiation*); however, if you intend to submit to *Acta Crystallographica Section C* or *E* or *IUCrData*, you should make sure that full publication checks are run on the final version of your CIF prior to submission.

Publication of your CIF in other journals

Please refer to the *Notes for Authors* of the relevant journal for any special instructions relating to CIF submission.

PLATON version of 06/05/2016; check.def file version of 05/05/2016

Datablock a064 - ellipsoid plot

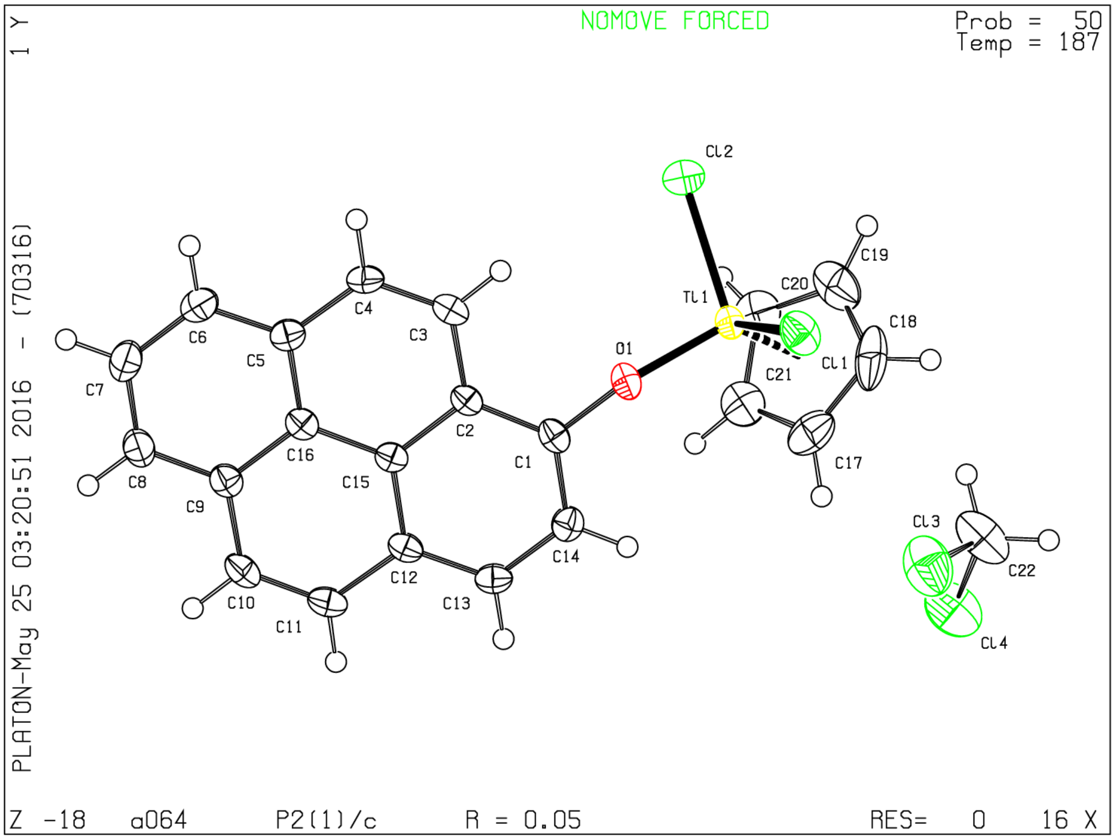

Supplement: Supplementary file 1 [file polymers-14-01427-s001.zip › Ti 3.pdf]
